# Observational birth cohorts for causal and predictive inference: The example of childhood asthma and allergic diseases

**DOI:** 10.1016/j.jaci.2025.03.005

**Published:** 2025-03-12

**Authors:** Brittney M. Snyder, Ewoud Schuit, Bryan S. Blette, William D. Dupont, Christian Rosas-Salazar, Karel K. G. Moons, Tebeb Gebretsadik

**Affiliations:** a Department of Medicine, Vanderbilt University Medical Center, Nashville;; b Department of Biostatistics, Vanderbilt University Medical Center, Nashville;; c Department of Pediatrics, Vanderbilt University Medical Center, Nashville;; d Julius Center for Health Sciences and Primary Care, University Medical Center Utrecht, Utrecht University, Utrecht.

**Keywords:** Birth cohort, asthma, allergic diseases, prospective studies, observational, causal inference, predictive modeling

## Abstract

Prospective birth cohort studies have identified important factors associated with the development and occurrence of early life conditions and facilitated exploration of causal mechanisms. We discuss the strengths, importance, and biases of birth cohort data for causal inference and predictive modeling, using childhood asthma and allergic disease research as an illustrative example. State-of-the-art study design and statistical methodologies are considered and recommended to mitigate bias and infer causality, as well as using cohort assembly for increased power, sample size, and generalizability. These include effective control for confounding, limiting loss to follow-up, and leveraging risk factors for precision. While logistical and methodologic challenges exist for establishing, maintaining, and analyzing birth cohorts and their respective data, this prospective study design offers numerous benefits for inferring causality over other observational designs, and it is often the only alternative for assessing critical research questions. With long-term follow-up and extensive data collection, birth cohort studies represent powerful tools for studying disease etiology and have been integral to developing effective treatment and prevention strategies. (J Allergy Clin Immunol 2025;155:1693–702.)

Birth cohorts are typically prospective, observational studies in which women and their offspring are recruited over a defined period (or time) and actively followed to allow measurement of exposures, outcomes, and other variables of interest.^1^ While recruitment and enrollment in birth cohorts has traditionally occurred during infancy, many cohorts are now enrolling prenatally, or even before conception, to aid in investigating developmental origins of health and disease.^2–4^ Offspring are then followed through childhood or adolescence with serial data collection, providing the opportunity to capture and characterize early life exposures with later life outcomes.^5^ Family and offspring characteristics, such as demographics, socioeconomic features, and lifestyle factors, and environmental exposures are often ascertained via self-reported questionnaires.^5^ Clinical data (eg, electronic medical records) and biological samples may also be linked and/or collected, creating a rich resource and vast data to address research questions of interest. Numerous birth cohorts have been established to identify *in utero* and early life causal and predictive factors for childhood diseases, such as asthma and allergic diseases, and health outcomes (Table I^6–12^; comprehensive list provided by Dubovyi et al^13^). These cohorts have identified important factors associated with the development and occurrence of these conditions in early life and facilitated exploration of causal mechanisms.^14^ For example, the seminal Tucson Children’s Respiratory Study (TCRS) demonstrated the association between the clinical phenotype of lower respiratory tract infection due to respiratory syncytial virus (RSV) and wheezing and asthma in early life.^15^ The Infant Susceptibility to Pulmonary Infections and Asthma Following RSV Exposure (INSPIRE) study identified that RSV infection in the first year of life, regardless of clinical phenotype, was associated with increased risk of childhood asthma development.^16^ Findings from the Childhood Origins of Asthma (COAST) and Copenhagen Prospective Study on Asthma in Childhood (COPSAC) birth cohorts have identified interaction effects between genetic variants within the highly replicated childhood asthma–related locus, 17q21, and rhinovirus, but not RSV, wheezing illnesses in the first year of life with respect to the risk of childhood asthma.^17^ In addition to asthma and allergic diseases, these birth cohorts, along with others, have been impactful in defining child lung function and, with repeat assessment in a subset of participants, the characterization of lung function growth through adolescence.^18^

Asthma and allergic diseases, including atopic dermatitis, allergic rhinitis, and food allergies, are common, chronic disorders affecting over 25% of children in the United States.^19,20^ These conditions are highly burdensome, with increased healthcare utilization and reduced quality of life among affected children and their families.^21,22^ Complex genetic and environmental interactions contribute to the heterogeneity of asthma and allergic diseases, resulting in variable age of onset and clinical presentation and lack of curative options.^14,23^ There is increasing evidence to suggest that these conditions have developmental origins, with prenatal and early life exposures invoking responses in biological programming of the fetus or infant which increase the risk of subsequent disease development.^4,5,23^ Much of this evidence comes from birth cohort studies.^23^ With long-term follow-up and extensive data collection, these studies represent powerful tools for studying disease etiology with the potential to inform development of effective treatment and prevention strategies (Fig 1).^14^

This review aims to discuss strengths, importance, and biases of birth cohort data for causal inference and predictive modeling and provide a state-of-the-art update on birth cohort methodologies and approaches in the context of childhood asthma and allergic disease research.

## STUDY DESIGN CONSIDERATIONS FOR INFERRING CAUSALITY IN BIRTH COHORTS

### Strengths of the birth cohort design for causal inference

Birth cohorts are often used to assess causal relationships between pregnancy and early life exposures and subsequent childhood health outcomes.^1^ This prospective study design offers numerous benefits for establishing causality over other observational designs, such as cross-sectional and case–control designs. Longitudinal data collection ensures that the exposure occurs prior to the outcome (ie, temporality) and that potential changes over time in exposure and variables of interest are also captured. With their prospective nature, birth cohorts allow for comprehensive measurement of exposures, outcomes, and other relevant information compared to retrospective studies (such as retrospective cohorts and case–control studies) where existing data are commonly used and may be incompletely, inaccurately, or inconsistently measured.^24^ Prospective cohort studies are also less prone to selection bias (excluding eligible participants in a way that skews effect estimates, including attrition) than case–control studies as the outcome is unknown at the time of enrollment, and as such, cannot affect who will and will not be included in the study.^25^

Randomized controlled trials (RCTs) are experimental studies in which participants are randomly assigned to intervention or control groups. These studies provide a higher level of evidence for causality than observational studies.^26^ However, RCTs are only suitable for some research questions. The experimental or randomized nature of RCTs limits their application to interventions for which there are reasonably strong expectations of improved health or social benefits.^5^ For some exposures, such as viral respiratory infection in infancy and neighborhood environmental exposures (eg, air pollution and proximity to major roadways), randomization is not possible or may be unethical.^5^ Additionally, RCTs are not suitable for identifying risk factors as these factors, unlike preventive or therapeutic interventions, cannot be randomized. RCTs are often expensive, making long-term follow-up difficult, and their performance among select populations often limits the generalizability of their findings.^5^ Additionally, children are often excluded from trials for numerous reasons, including the biological heterogeneity of this population (eg, prepubertal children are different from postpubertal adolescents and young adults) and the small market for drugs and devices within this population as children are generally healthy.^27^ Similarly, pregnant women are rarely studied in trials for reasons including potential harm to the fetus.^28^ The limitations of RCTs highlight the need for observational study designs, such as birth cohorts, which are often the only alternative for assessing important research questions.

### Potential challenges for causality using birth cohorts

Birth cohorts are associated with logistical and methodologic challenges. Logistically, designing, establishing, and maintaining birth cohorts is expensive and time-consuming. Additionally, with long follow-up periods, participant attrition is of concern. It is important to implement strategies to minimize loss to follow-up^29,30^ as with lower rates of follow-up, validity is reduced as systematic differences in the outcome and/or exposure may exist between those who remain in the study and those who were lost to follow-up.^24^ Birth cohorts are more amenable to assessing childhood outcomes than outcomes that occur later in life. However, the necessary time between exposure and outcome development increases the likelihood of confounding (common cause of exposure and outcome).^5^ Vulnerable populations are often disproportionately affected by adverse exposures and diseases; however, there are challenges to enroll and maintain these populations in birth cohorts. As such, multiple approaches should be taken to maintain diverse representation in birth cohort studies.^2^

Methodologic issues that may limit the ability to establish causal relationships in observational research include reverse causation (outcome affects the exposure), confounding, selection bias, and information bias or measurement error, including bias due to self-report.^5,31^ The design of birth cohort studies minimizes some of these problems. Prospective data collection reduces the likelihood of reverse causation (eg, temporal sequencing [cause precedes the effect]) and selection bias (eg, recall or information bias) as timing of the exposure and outcome are known and the outcome is unknown at the time of enrollment, respectively.^5,25^ Selection bias may also be reduced through design considerations aimed at limiting loss to follow-up (eg, calling participants for questionnaire completion rather than sending questionnaires via mail) and analytic techniques that account for missing data due to loss to follow-up (eg, inverse probability weighting^32^ for selection bias and multiple imputations for covariates).^5,33^ Information bias and measurement error can be reduced through protocolized and standardized repeated measurement of exposures, outcomes, and confounders.^5^ Numerous design and analytic strategies have been developed to mitigate biases and confounding in birth cohorts and strengthen causal inference.^34^ Several of these strategies will be discussed in later sections.

### Nested study designs for inferring causality

Once a birth cohort is established, several complementary study designs for inferring causality (ie, nested designs) may be employed using cohort data. These designs benefit from collection of a reduced number of study subjects and corresponding reduction in costs of data collection while maintaining statistical efficiency that is comparable to the full cohort study.^35,36^ Examples of nested designs include nested case–control studies, case-cohort studies, and self-controlled studies. While this section focuses on the more commonly used design in biomarker studies (nested case–control), case-cohort and self-controlled designs have been extensively reviewed elsewhere.^37,38^

Nested case–control studies include participants with (cases) and without (controls) the outcome of interest selected from those at risk for the outcome within the original cohort but who are outcome-free when the case participant is diagnosed. This design is particularly useful when data collection is expensive (eg, genomic analysis) or invasive and the disease outcome is rare in that costly and time-consuming data need only be collected from cases and selected controls.^36,39,40^ An example of a study utilizing a nested case–control design within a birth cohort to answer an asthma-related causal question is provided by Shrestha et al,^41^ who determined the association of inhaled corticosteroid use with pneumonia risk (ie, rare outcome) among children with asthma enrolled in the Mayo Clinic Birth cohort. Cases were defined as those with pneumonia on the basis of validated International Classification of Diseases codes, confirmed through manual medical chart review. Controls were selected from those who did not develop pneumonia and were matched 1:1 with cases on the basis of sex, age, and asthma index date (±1 year).

## STATISTICAL CONSIDERATIONS FOR INFERRING CAUSALITY IN BIRTH COHORTS

### Relationship of variables

Confounding is a major threat to causal inference in observational studies, including birth cohort studies. Because the exposure is not randomized in birth cohorts, differences in measured and unmeasured confounders between comparison groups usually exist and may bias results.^34^ Confounders are variables related to the exposure and the outcome and are not on the causal pathway between the exposure and the outcome (ie, intermediate variables). Careful control of confounding, while challenging, is an essential step in the conduct of observational causal studies.^42^

#### Directed acyclic graphs.

The substantial amount of data collected within, and potentially linked to (see later section on birth cohort augmentation), birth cohorts is not only useful for examination of numerous exposure-outcome relationships, but it is also important to account for potential factors that may confound these relationships. While it is helpful to have measurements on several potential confounders available for inclusion in statistical models, it is generally not advisable to include all measured variables in a statistical model due to increased complexity and the potential for collider bias, over-adjustment, or reduced regression power or spurious results due to overfitting.^43–45^ Confounder selection via a directed acyclic graph (DAG) or prespecification with clinical or background knowledge can increase interpretation of reported associations.^43,46^ It is important to carefully consider potential confounders for a multitude of potential causal questions when establishing a prospective birth cohort.

**FIG 2.**
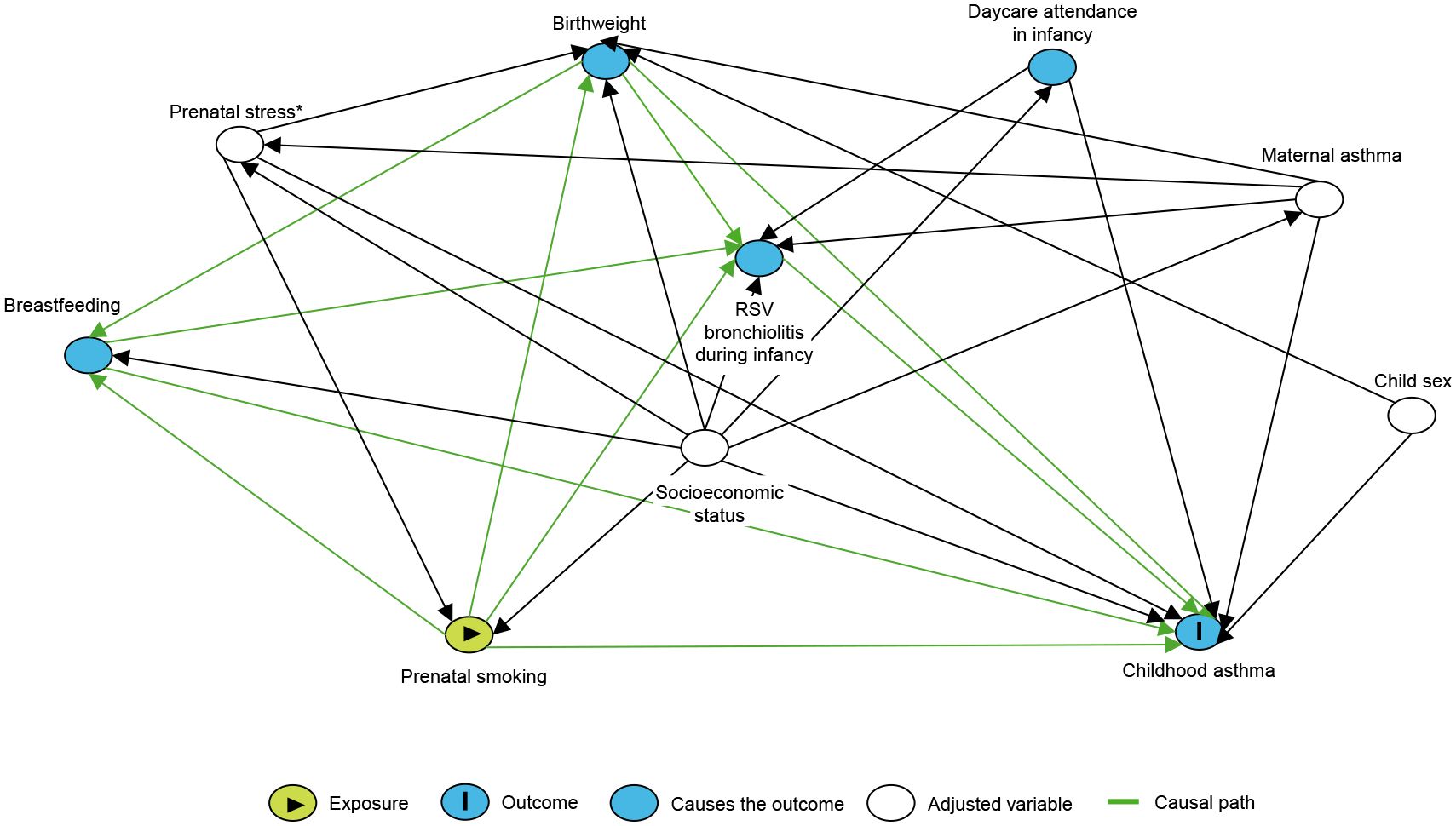
DAG examining causal relationship between prenatal smoking and childhood asthma. Created by DAGitty.net. *Prenatal stress is considered confounder in this DAG. However, relationship between prenatal stress and prenatal smoking may be more complex than what is depicted—that is, prenatal stress may be on causal pathway from prenatal smoking to childhood asthma. Therefore, different relationships should be considered in sensitivity analyses.

DAGs visually summarize causal relationships between variables, where arrows between variables specify the existence and direction of a causal relationship, but not the magnitude, sign, shape, or form.^46^ The acyclic nature of DAGs intuitively implies that variables cannot be caused by themselves.^46^ Drawing and sharing a DAG allows investigators to demonstrate their theories about relationships relevant to their causal research question. It also makes these assumptions transparent and open for scrutiny from the scientific and clinical community.^46^ An example DAG examining the causal relationship between prenatal smoking and childhood asthma is provided in Fig 2. By adjusting for 4 variables for which data are available (ie, measured) in the example DAG, prenatal stress, socioeconomic status, maternal asthma, and child sex, bias is minimized when estimating the causal effect of the exposure (prenatal smoking) on the outcome (childhood asthma), assuming the causal assumptions provided in the DAG hold.^47^ Several important relationships are observed in this graph. First, a confounder is present, socioeconomic status, which influences exposure and predicts outcome. Second, child sex is captured, which is a factor predictive of outcome but without known influence on the exposure. It is recommended to adjust for such factors, in addition to measured confounders, in statistical models to increase efficiency of the estimated exposure effect without increasing bias.^48^ Third, an intermediate variable is observed, breastfeeding, which lies on the causal pathway between the exposure of interest and the subsequent outcome (ie, prenatal smoking is associated with a lower likelihood of breast-feeding,^49^ and not breastfeeding is subsequently associated with increased risk of childhood asthma^50^). Outside of formal mediation analysis, intermediate variables should be considered with caution for adjustment in statistical models as this may induce bias, usually towards the null, by masking the effect of the exposure on the outcome (sensitivity analyses may be carried out to gauge their influence).^48^ Fourth, collider variables are observed, birthweight and RSV bronchiolitis during infancy, resulting from two independent causes having a common effect. Colliders should not be conditioned on in study design or included as covariates in adjusted statistical models as this would incorrectly induce an association between two previously independent causes.^48^ For example, if birthweight is included in the statistical model, bias could be introduced by generating a spurious association and incorrectly opening a backdoor path that did not previously exist between prenatal stress and child sex. Collider bias can also attenuate or hide underlying true associations.^51^ Additionally, low response rates or differential loss to follow-up can lead to collider bias by limiting to a population subgroup. It is important to realize that in a DAG model, all causal connections are specified and that variables that are not linked (via an arrow) are not causally connected. In Fig 2, for example, it is assumed that there is no causal association between prenatal smoking and child sex.

#### Additional variables for consideration in model adjustment when assessing causal relationships.

**FIG 3.**
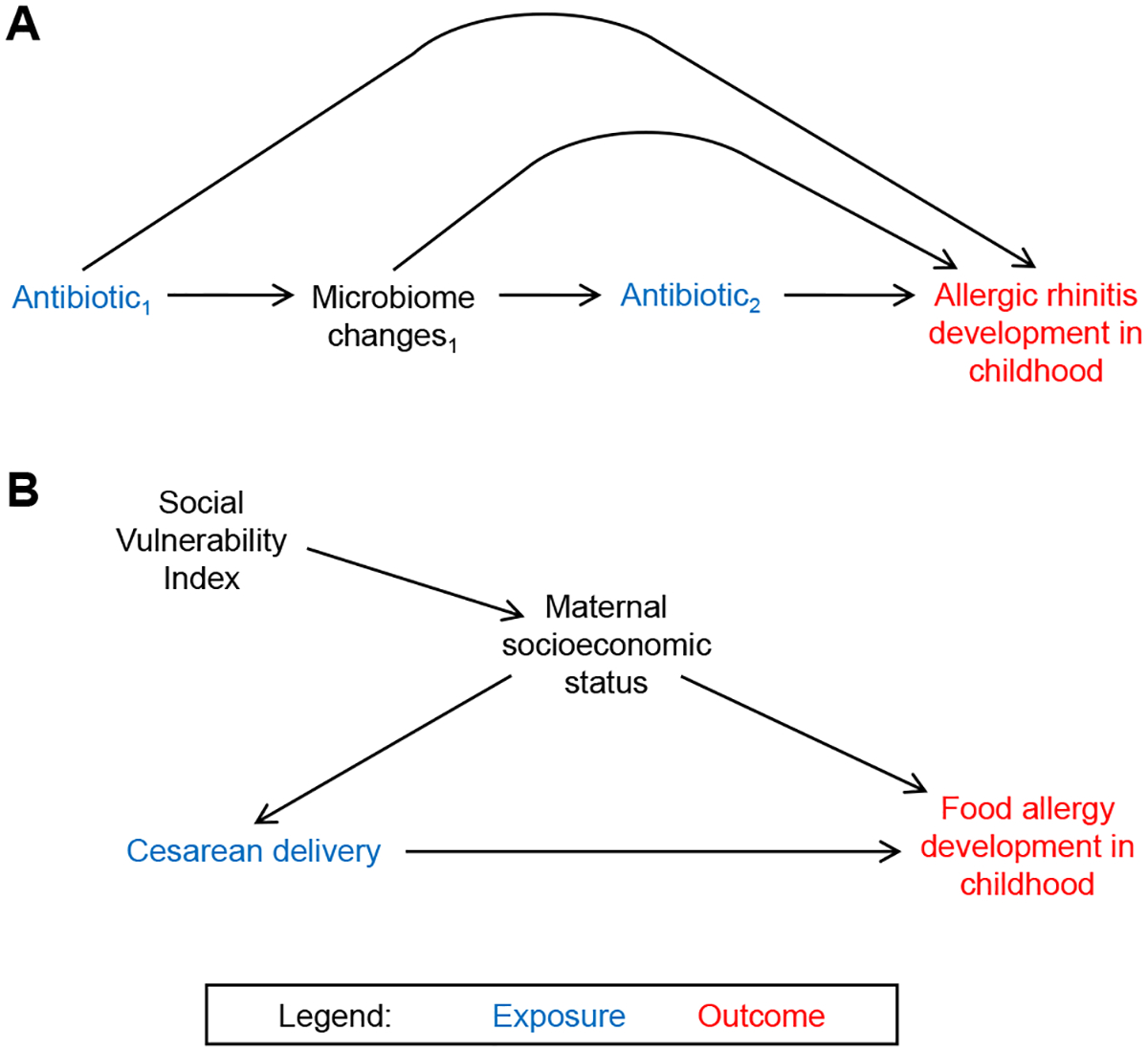
Example of **(A)** time-varying confounding and **(B)** proxy and latent variables. *(A)* Antibiotic receipt (exposure) affects microbiome (time-varying confounder), leading to additional receipt of antibiotics. Advanced statistical methods must be used to properly estimate effect of antibiotic receipt on allergic rhinitis development in childhood (outcome) using longitudinal data. *(B)* In assessing causal relationship between cesarean delivery (exposure) and food allergy development in childhood (outcome), proxy variable that could be used in place of direct measurement of maternal socioeconomic status (latent variable) is SVI. However, SVI is neighborhood-level variable and may not be as precise as individual-level variables such as maternal education or income level.

Other important factors to consider when selecting variables for model adjustment in a causal research study are time-varying confounders and proxy, latent, and instrumental variables (the latter of which is discussed in the statistical methods section). Confounders with values that change over time are referred to as time-varying and often co-occur with time-varying exposures (Fig 3, *A*). When time-varying confounding is present, confounders are affected by past exposure and influence future exposure, and longitudinal data on both the exposure and time-varying confounders are needed for adequate effect estimation. Conventional statistical methods, such as regression modeling, are inadequate in controlling for these types of confounders. More advanced methods, such as inverse probability of treatment weighting, must be used to properly estimate the effect of a time-varying exposure on an outcome using longitudinal data.^42^ Details of these methods are beyond our scope here and have been reviewed elsewhere.^52^

Proxy variables are measured variables that can stand in for unmeasured variables that are difficult or impossible to measure directly.^48^ In assessing the causal relationship between cesarean delivery and food allergy development in childhood (Fig 3, *B*), a proxy variable that could be used in place of direct measurement of maternal socioeconomic status (confounder) is the Social Vulnerability Index (SVI).^53^ Maternal socioeconomic status is also a latent variable representing an underlying construct that cannot be measured directly and must be inferred from measured variables. Adjusting for proxy or latent confounder variables when information on the actual confounders or confounder mechanisms is unavailable can reduce bias in the unadjusted, or crude, effect estimate.^48^ Race and ethnicity are social constructs that are sometimes used as proxy measures for exposure to experiences of systemic racism.^54,55^ While limitations to these terms exist, their inclusion in birth cohort research is important for addressing and elucidating health inequities and disparities. In-depth discussion on appropriate use and reporting of these variables is outside the scope of this review; however, detailed guidance is provided by the *Journal of the American Medical Association*.^54^

Additional ways to limit confounding bias in a birth cohort study’s research question-specific design phase include matching, stratification, and restriction. Using the example provided in Fig 2, socioeconomic status confounds the relationship between prenatal smoking and childhood asthma. Confounding by socioeconomic status could be reduced by restricting the cohort to either individuals of lower or higher socioeconomic status, matching individuals on the basis of their socioeconomic status, or stratifying (dividing) the cohort into socioeconomic status–specific subgroups and performing subgroup-specific analyses. While useful, limitations of these methods, such as potential for reduced sample size due to exclusion of individuals who do not meet matching criteria and loss of generalizability,^56^ must be considered and findings should be interpreted cautiously.

### Augmenting birth cohort data with physical and social environmental data

Environmental exposures, including physical and social exposures, play an important role in health and disease, particularly asthma and allergic diseases, and can contribute to health disparities.^57^ An individual’s physical environment includes both natural (eg, water and air quality) and built (eg, buildings and roads) environments,^58^ while their social environment includes interpersonal relationships within their family, friend groups, neighborhoods, and the social system and structure.^59^ To facilitate a more comprehensive assessment of environmental, geographic, sociodemographic, and community-level exposures, linkage of participant residential addresses with physical and social environmental records is increasingly being performed to supplement originally collected birth cohort data. These linkages also allow better capture of health inequity exposures and/or confounders that may affect health. Physical environmental data, including US Environmental Protection Agency air and water quality data^60^ and the Smart Location Database^61^ summarizing more than 90 indicators associated with the built environment and location efficiency (eg, street network design, demographic and employment statistics, and density of land development), are publicly available.

Several tools and indices have been developed to aid researchers in assessing social determinants of health and identifying vulnerable communities, each with their own goals and focus.^62^ Publicly available neighborhood-level social indices include the SVI^53^ and Childhood Opportunity Index.^63^ However, an inherent limitation of these indices lies in their specificity to geographical areas such as the United States with built-in infrastructure for linkage with environmental and/or social data.^64,65^

### Statistical methods for causal modeling

This section reviews key terminology, assumptions, and statistical techniques in formal causal inference, aligned with innovation in causal inference methods and a focus on observational study data availability.

#### Assumptions and conditional causal effects.

Standard assumptions for drawing causal inference include:^66–71^

1. The stable unit treatment value assumption has two main components for ensuring the treatment effect is well defined and can be accurately estimated:
  - Noninterference: The exposure of one individual does not affect the outcome of another individual.
  - Consistency: The observed outcome for an individual under the exposure or treatment of interest is the same as the potential outcome that would be observed if that treatment had been assigned.
2. Exchangeability: No unmeasured confounding or no hidden factors influencing the results. The potential outcomes of exposed and unexposed individuals are independent of the exposure they received, conditional on measured confounders.
3. Positivity: Participants, regardless of their characteristics, have at least some chance of receiving the exposure.

These concepts are presented under the causal framework to highlight their importance. However, the need to meet assumptions will depend on study design and research questions, such as comparative treatment effectiveness studies and dealing with confounding by indication. Although further detail is beyond the scope of this review, referral to the provided landmark references is recommended for examples and guidance on their application.^66–71^ Under these assumptions and time-fixed confounding, conventional multivariable regression modeling with *a priori* knowledge informing confounder adjustment, is a powerful tool for estimating causal effects. The estimated exposure coefficient in an adjusted model is a consistent estimator of the causal effect of the exposure on the outcome, conditional on other variables included in the model. For example, consider a birth cohort study investigating the effectiveness of a new topical treatment for atopic dermatitis in children. The investigators fit a model adjusted for age, including an interaction between treatment and age. They find that the treatment is generally effective (ie, the treatment reduces atopic dermatitis flares), but its effectiveness is significantly higher in infants than older children. Here, the estimated causal effect of the topical treatment on atopic dermatitis is conditional on and differs by participant age (also known as an effect modifier).

#### Understanding marginal causal effects in regression models.

Often marginal causal effects, or effects not conditional on other variables in the regression model, are of interest. Within the context of the example above, researchers may be interested in calculating the marginal effect of increasing the dosage of the topical treatment by one unit (eg, 1 mg), which would show how much the probability of symptom improvement changes with each additional unit of treatment assuming all other variables included in the model remain constant. This effect represents the average impact of the treatment across the study population, regardless of age and other characteristics. For linear regression models (ie, where *Y*, response outcome, is measured continuously) with no interactions between exposure and confounders, the estimated exposure coefficient targets conditional and marginal causal effects. However, for models where the outcome is binary or time to event and models with interactions, more complex methods, such as standardization,^68^ are needed to estimate a marginal causal effect.

#### Propensity scores and inverse probability methods.

Another popular set of methods for estimating marginal causal effects uses propensity scores. Propensity scores are the conditional probability of each individual being exposed given their confounder data. These can be used in several ways to estimate marginal causal effects. Propensity score matching entails estimating a propensity score for each cohort participant (often using a regression model) and matching control individuals to exposed individuals to minimize differences in their matched propensity scores.^72^ A comparison of the outcomes between exposed individuals and their matched controls estimates the marginal causal effect of the exposure on the outcome. A second approach involves adjusting for the estimated propensity score in a multivariable regression model and then taking the estimated coefficient for exposure as the estimated causal effect.^73^ A third approach weights each individual by their inverse probability of exposure (inverse probability of treatment weighting) and then fits a simple weighted model relating the outcome to the exposure.^74^

#### Instrumental variables and Mendelian randomization.

The rich longitudinal data collection undertaken in birth cohorts makes the exchangeability assumption of no unmeasured confounding in causal analyses more plausible than in other observational designs. However, in many scenarios, this assumption remains implausible. In these settings, a separate set of assumptions can be used to draw causal inferences using an instrumental variable. An instrumental variable is a variable that causes the exposure but has no effect on the outcome except through its effect on the exposure.^48,75^ These methods also assume that there is no causal relationship between the instrumental variable and any measured or unmeasured confounders and that the association between the exposure and instrumental variable is relatively strong. The most common approaches to estimating causal effects are 2-stage least squares, where one regresses the exposure on the instrumental variable and then regresses the outcome on the fitted values for the exposure. However, depending on the nature of the outcome variable, other methods such as maximum likelihood estimation, 2-stage residual inclusion, or generalized method of moments may be more appropriate, particularly for binary or nonlinear outcomes.^76–78^

**FIG 4.**
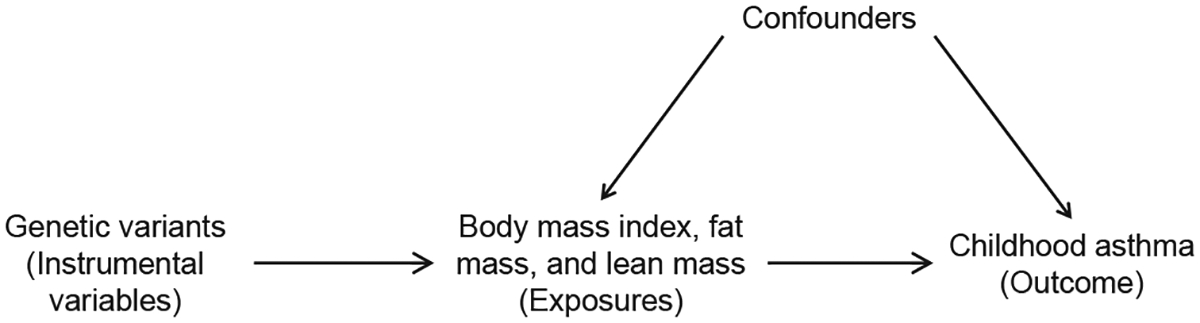
Example of Mendelian randomization in context of childhood asthma. Genetic variants act as instrumental variables in association between adiposity and childhood asthma as they are related to body mass index, fat mass, and lean mass (exposures) but not childhood asthma (outcome). This is a simplistic depiction of Mendelian randomization, and it is important to recognize strong assumptions that must be met when utilizing this method—for example, genetic variants are associated with exposure, genetic variants are only associated with outcome through exposure (exclusion restriction assumption), and genetic variants are independent of other factors that affect outcome (independence assumption).

Mendelian Randomization is an example of a framework which uses an instrumental variable strategy (Fig 4).^79^ In this design, genetic variants that are associated with the exposure act as instrumental variables. As genetic associations are considered free from confounding, differences in outcomes between genetically defined groups can be attributed to the exposure when the genetic variants affect exposure but no other phenotype.^80^ However, it is important to recognize that genes can have pleiotropic effects on phenotypes. If a phenotype (exposure) that is related to the genetic variants of interest is unknown but is causally linked to the outcome of interest, the genetic variants cease to be instrumental variables and regressing outcome against genotype can result in a spurious relationship between the known phenotype and the outcome.^81^

In settings where a valid instrumental variable cannot be identified, negative control exposures or negative control outcomes can also be leveraged to estimate causal effects under certain types of unmeasured confounding.^82^ Negative control exposures and negative control outcomes are variables that do not affect the outcome or are unaffected by the exposure of interest, respectively. If an association between the negative control exposure and an outcome, or an exposure and an negative control outcome, is found, this is evidence of residual confounding bias. Using the example of a birth cohort study investigating the effectiveness of a new topical treatment for atopic dermatitis in children, an arm fracture could be used as an negative control outcome as it is unrelated to atopic dermatitis or its treatment. Finding no association between the topical treatment and an arm fracture, as expected, would increase confidence in the observed causal effect of the topical treatment on atopic dermatitis.

## BIRTH COHORT DATA FOR PREDICTIVE INFERENCE

In addition to causal inference, birth cohorts can be used for prediction studies.^83^ By combining information from multiple characteristics of an individual (predictors that include, but are not limited to, demographics, medical history, lab measurement, and environmental factors) into a multivariable model, it is possible to estimate an individual’s risk of having (diagnosis) or future occurrence (prognosis) of an outcome of interest.^84^ Once validated, these models can be used in the clinical setting for risk assessment (allowing for early detection and prevention measures), treatment planning, improved healthcare provider-patient communication and shared decision-making, and resource allocation (identifying high-risk patients for effective prioritization of care and resources).^85^

Typically, predictive modeling is considered descriptive because there is no primary interest in causal associations between individual predictors and the outcome, but instead in the combination of predictors that best predicts the outcome.^83^ Because of this, confounding is less of an issue in prediction, compared to causal, research. However, it is important to be aware of strong causal factors (eg, via consultation with experts or review of scientific literature) when selecting candidate predictors to include in the model as the best predictors are causally associated with the outcome. Assessing generalizability (ie, model performance in the target population from which the study population was selected) and transportability (ie, model performance in a population outside the target population) of the developed prediction model is essential for valid inference and clinical implementation.^86–89^ Examples of clinical prediction indices and models for assessing childhood asthma risk include the Asthma Predictive Index^90^ and the modified Asthma Predictive Index^91^ and the Pediatric Asthma Risk Score.^92^

Prediction models can be assessed for their risk of bias, or systemic distortion of predictive performance due to deficiencies in study design, conduct, or analysis using PROBAST (Prediction Model Risk of Bias Assessment Tool; www.probast.org).^93,94^ PROBAST is organized into 4 domains (participants, predictors, outcome, and analysis), including a total of 20 signaling questions for judgment of a study’s risk of bias.^93^ Although many signaling questions, mainly in the analysis domain, are independent of study design, using a prospective birth cohort will positively affect a model’s risk of bias in the participant, predictor, and outcome domain due to design strengths (described in the former section on study design considerations).

When the aim is to develop or validate a prediction model from a birth cohort study dataset, it is essential to pay attention to all aspects that can potentially lead to bias (eg, by using PROBAST for guidance). This includes, for example in the outcome domain, determining the outcome ideally without knowledge of the predictors and preventing incorporation of predictors in the outcome definition. In general, following the most recent guidance on model development and validation^95–97^ is recommended,^98^ investigating validity (eg, using bootstrapping techniques or cross-validation), assessing relevant model performance measures (eg, calibration and discrimination^99^), and adequate reporting (eg, presenting model coefficients to allow readers to estimate risks for individual participants).^95^

## CONCLUSIONS

We have highlighted the vast array of potential of birth cohort data for assessing associations, illustrated with childhood asthma and allergic disease research. Best practices and methodologies were reviewed, including design and statistical considerations for causal modeling and using birth cohort data for predictive inference. While logistical and methodologic challenges exist for establishing, maintaining, and analyzing birth cohorts and their respective data, this prospective study design offers numerous benefits for inferring causality over other observational designs. It is often the only alternative for assessing critical research questions, such as determining associations between exposures that cannot be randomized (eg, viral respiratory infection in infancy or neighborhood environmental exposures) and the development of childhood asthma and allergic disease. Many factors and predictors for disease development have been identified through birth cohort studies, contributing to multi-level support for causal and predictive roles of these factors which has been integral in informing both therapeutic and preventive interventional studies.

## Figures and Tables

**FIG 1.**
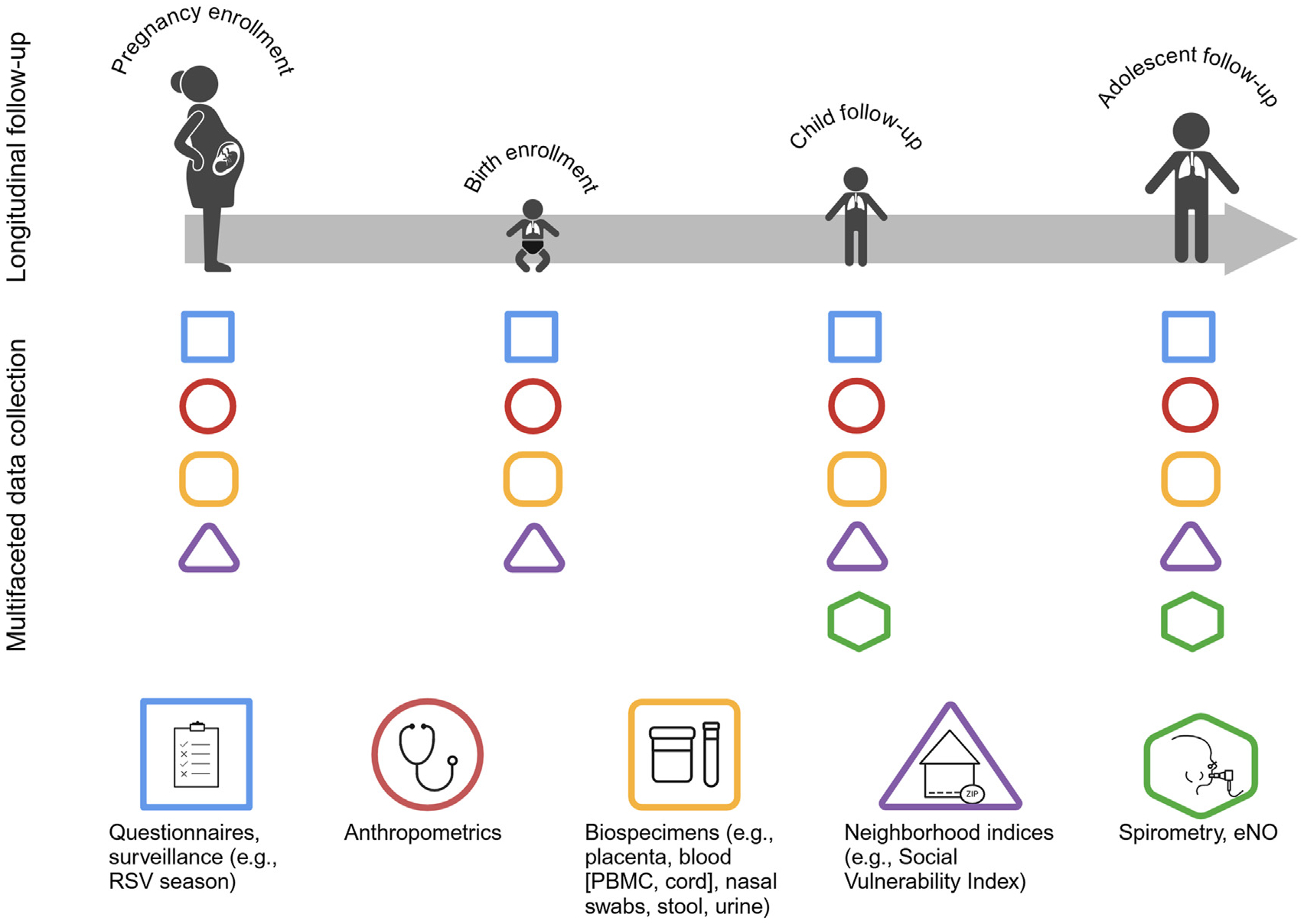
Prospective birth cohorts are powerful tools for studying etiology of childhood asthma and allergic diseases. Created by BioRender.com. *eNO*, Exhaled nitric oxide; *PBMC*, peripheral blood mononuclear cell.

**TABLE I.** Example birth cohorts, by decade of enrollment, established to identify *in utero* and early life factors for childhood asthma and allergic diseases

Decade of enrollment	Cohort	Location	Population	Recruitment start year	Initial enrollment number	Reference
1980	TCRS	Tucson, Ariz	General	1980	1,246	6
1990	COAST	Madison, Wis	High risk: At least one parent with allergies and/or asthma	1998	287	7
	COPSAC	Copenhagen, Denmark	High risk: Mothers with asthma	1998	411	8
2000	CCAAPS	Cincinnati, Ohio	High risk: At least one parent with atopy	2001	680	9
	URECA	New York, NY; St Louis, Mo; Baltimore, Md; Boston, Mass	High risk: At least one parent with allergic disease and/or asthma	2005	560	10
2010	INSPIRE	Middle Tennessee	General	2012	1,952	11
2020	CANOE	Detroit, Mich; St Louis, Mo; Madison, Wis; Nashville, Tenn	High risk: At least one biological parent or sibling with asthma, allergic rhinitis, and/or atopic dermatitis	2020	573	12

*CANOE*, Childhood Allergy and the Neonatal Environment; *CCAAPS*, Cincinnati Childhood Allergy and Air Pollution Study; *COAST*, Childhood Origins of Asthma; *COPSAC*, Copenhagen Study on Asthma in Childhood; *INSPIRE*, Infant Susceptibility to Pulmonary Infections and Asthma Following RSV Exposure; *TCRS*, Tucson Children’s Respiratory Study; *URECA*, Urban Environment and Childhood Asthma.

## Abbreviations used

DAG: Directed acyclic graph
PROBAST: Prediction Model Risk of Bias Assessment Tool
RCT: Randomized controlled trial
RSV: Respiratory syncytial virus
SVI: Social Vulnerability Index
